# Gut-directed hypnosis and hypnotherapy for irritable bowel syndrome: a mini-review

**DOI:** 10.3389/fpsyg.2024.1389911

**Published:** 2024-06-03

**Authors:** Winfried Häuser

**Affiliations:** ^1^Department of Psychosomatic Medicine and Psychotherapy, Technical University Munich, Munich, Germany; ^2^Medical Center Pain Medicine and Mental Health, Saarbrücken, Germany

**Keywords:** irritable bowel syndrome, gut-directed hypnosis, efficacy, gastroenterology, guidelines, digital health applications

## Abstract

Irritable bowel syndrome (IBS) is a frequent health condition which can be associated with functional disability and reduced health-related quality of life. IBS is classified as a disorder of the brain-gut axis. IBS is a very heterogenous condition with regards to the underlying pathophysiological mechanisms, the clinical picture and the amount of functional impairment. Within a biopsychosocial model of IBS psychosocial factors can play a role in the in the predisposition, triggering and development of chronicity. Somatic or psychosocial or a mixture of both factors might predominate in an individual patient. Gut-directed hypnosis is a special type of medical hypnosis combining standardised gut-directed suggestions (hypnosis) with suggestions tailored to the psychological characteristics of the patient (hypnotherapy). Of brain-gut behavioral therapies, cognitive bahvioral-based interventions and gut-directed hypnosis have the largest evidence for both short-term and long-term efficacy in controlled trials for IBS and are recommended by current European and North American gastroenterology guidelines as second line treatment options. Standardised gut-directed hypnosis is available by audiotapes and can be part of a multicomponent self-management approach by digital health applications. It can be used – based on the patient‘s preferences—as first line therapy for mild forms of IBS. Severe forms of IBS require face-to-face interdisciplinary management. Standardised gut-directed hypnosis and hypnotherapy tailored to the individual patient can be part of this approach.

## Introduction

Gastrointestinal symptoms such as abdominal pain, stool and defecation problems, and bloating are extremely common in the general population at any particular point in time and are experienced on a continuum, from short self-limiting to clinical conditions with negative impact on social functioning, and health-related quality of life. In up to 50% of persons with chronic abdominal symptoms seeking for medical care, no somatic disease sufficiently explaing the symptoms can be found (Enck et al., 2016). Different approaches with regards to diagnostic criteria are used by different medical specialties for these medical conditions. They are labeled “medically unexplained somatic symptoms” in general medicine, functional somatic syndromes in internal and psychosomatic medicine and “somatoform disorders” or “bodily distress syndrome” in psychiatry and psychology (Burton et al., 2020).

Hypnosis has a long history of ups and downs of its role in somatic medicine. In the era of evidence-based medicine, the importance of a treatment depends on the availabilty of studies demonstatring its efficacy and effectiveness and its inclusion in clinical guidelines (Häuser et al., 2016; Häuser, 2022; Peter, 2023).

This mini-review will focus of one of the most frequent medical conditions associated with abdominal symptoms, namely irritable bowel syndrome (IBS) and the significance of hypnosis in its management. The aims of the paper are as follows:

- To give an overview on the diagnostic criteria of IBS
- To discuss the importance of psychological therapies in general and of gut-directed hypnosis in particular as outlined in gastroenterology guidelines in the management of IBS
- To give an overview on the techniques, the mechanisms of action and the efficacy/effectiveness of gut-directed hypnosis for IBS

### IBS-diagnostic criteria

IBS can be defined by the criteria of the Rome Foundation, an independent not-for-profit organization. The most recent iteration, Rome IV, were published in 2016 These define IBS as the presence of abdominal pain, related to defaecation, associated with a change in stool frequency and/or stool form. Patients are subgrouped according to their predominant stool pattern into IBS with diarrhoea (IBS-D), IBS with constipation (IBS-C), IBS with mixed bowel habits (IBS-M) or IBS unclassified (IBS-U), to direct therapy (Drossman and Hasler, 2016). This subgrouping has been driven by the need to create subgroups for specific pharmacological therapies. The Rome-IV subgrouping neglects one of the most embarassing for patients, namely stool urgency (Saha, 2014) as well avoidance behaviours (food, sexual activities, school/work, leisure times) of patients (Häuser, 2022).

To exclude a somatic disease, current guidelines recommend some obligatory baseline investigation (e.g., blood and stool tests) and – depending on the main symptom (e.g., diarrhea, constipation) and the age of the patient—additional specialist diagnostic (e.g., colonoscopy) (Lacy et al., 2021; Layer et al., 2021; Vasant et al., 2021).

As in somatic diseases such as heart insufficiency or liver cirrhosis, different degrees of IBS-severity in IBS can be differentiated. Severity can be defined by a biopsychosocial composite of patient-reported gastrointestinal and extraintestinal symptoms, degree of disability, and illness-related perceptions and behaviors. Severity can be subcategorized into clinically meaningful subgroups as mild (∼40%), moderate (∼35%), and severe (∼25) (Drossman et al., 2011).

### Irritable bowel syndrome – a disorder of gut-brain interaction

IBS is a very heterogenous condition with regards to the main gastrointestinal symptoms, the amount of associated other somatic symptoms, psychological distress and disability. The pathophysiological mechanisms vary between the patients (Ford et al., 2020). IBS is conceptualised as a disorder of altered bidirectional communication between the gut and brain (via the gut-brain axis). It has a biopsychosocial aetiology: Genetics, and epigenetic changes, infection and early adverse life events may predispose an individual to developing IBS. Chronic stress, mental disorders (anxiety, depression), negative beliefs about symptoms and maladaptive coping mechanisms can increase the frequency and severity of symptoms (Layer et al., 2021; Vasant et al., 2021). Therefore, the Rome IV process redefined IBS, formerly called functional gastrointestinal disorders, as a disorder of gut-brain interaction, in recognition of the complex interplay of biological, psychological, and social factors underpinning the condition (Drossman and Hasler, 2016).

### Management of IBS

There is no known cure for IBS and treatment is limited to symptom management strategies. Current guidelines (Germany, Great Britain, United States) (Lacy et al., 2021; Layer et al., 2021; Vasant et al., 2021) recommend a graduated and tailored (according to symptoms) management approach (see Table 1). First and second line pharmacological treatments are recommended for primary and secondary care (respectively) (see Table 1).

**Table 1:** Graduated treatment approach of irritable bowel syndrome [adapted from Drossman and Thompson (1992)].

Step 1 (all patients, might be sufficient for mild forms of IBS): education, reassurance, promotion of healthy life style and dietary adjustment
Step 2 (moderate IBS): pharmacological agents according to the main symptom (pain, diarrhea, constipation). Diet. Psychological therapies
Step 3 (severe IBS): multicomponent and interdisciplinary treatment including and psychopharmacologic agents and combined psychological treatments

### The importance of psychological therapies in the management of IBS

Making a positive diagnosis, information on normal life expectancy, explanation of the condition within an individual biopsychosocial model based on the history and findings of the patient, discussing treatment options based on the previous experiences and preferences of the patient, managing expectations and enhancing self-efficacy of the patient by promoting a healthy life-style are the basic psychological actions to be taken by the primary care physician and the gastroenterologist (Lacy et al., 2021; Layer et al., 2021).

Psychological therapies are recommended as second line treatment when symptoms /disability will not improve after three months (Layer et al., 2021) and 12 months therapy, respectively (Vasant et al., 2021). The British guideline states that referral can be made at an earlier stage, if accessible locally, and based on patient preference (Vasant et al., 2021). The German guideline recommends psychological treatments in case of significant disability and of mental comorbidities and /or dysfunctional coping (Layer et al., 2021).

### Gut-directed hypnosis of IBS – mechanisms of action, efficacy, and effectiveness

Several psychological therapies are efficacious for IBS, although none are superior to another. CBT-based interventions and gut-directed hypnotherapy have the largest evidence for both short-term and long-term efficacy in RCTs (Black et al., 2020).

Gut-directed hypnosis modulates the gut-brain axis, with several studies demonstrating positive changes in gut-brain function before, and immediately after, hypnosis, including modulation of postprandial gastro-colic reflex activity, altered colonic motility, reduced visceral hypersensitivity and normalisation of gut-brain pain processing signals on functional brain imaging (Vasant et al., 2021). It may alter the patient’s focus of attention and/or his/her beliefs about the meaning of sensations from the gastrointestinal tract, because other somatic symptom and psychological distress are reduced after treatment (Palsson et al., 2002).

The efficacy of gut-directed hypnosis has been demonstrated with the highest level of evidence, namely systematic reviews of randomised controlled trials. Eight randomized controlled trials with a total of 464 patients and a median of 8.5 (7 to 12) hypnosis sessions over a median of 12 (5 to12) weeks were included into one systematic review. At the end of therapy, hypnosis was superior to control conditions in producing symptom relief (Relative Risk [RR], 1.69; 95% Confidence Interval [CI] 1.14 to 2.51), Number needed to treat [NNT] 5 [95% CI 3 to 10] and in reducing global gastrointestinal score (Standardised Mean Difference 0.32 [95% CI 0.56 to 0.08]). At long-term follow-up, hypnosis was superior to controls in adequate symptom relief (RR, 2.17 [95%CI 1.22 to 3.87]; NNT 3 [2 to10]) (Schaefert et al., 2014). Another meta-analysis of 6 RCTs, recruiting 639 patients, reported a RR of remaining symptomatic of 0.73 (95% CI 0.55 to 0.97) compared with education and/or support and 0.67 (95% CI 0.49 to 0.91) compared with a waiting list control (Black et al., 2020). In addition, there are convincing long-term outcome data demonstrating effectiveness in routine clinicla care. 204 patients prospectively completed questionnaires scoring symptoms, quality of life, anxiety, and depression before, immediately after, and up to six years following hypnotherapy and assessed the effects of hypnotherapy retrospectively in order to define their “responder status.” 71% of patients initially responded to therapy. Of these, 81% maintained their improvement over time while the majority of the remaining 19% claimed that deterioration of symptoms had only been slight (Gonsalkorale et al., 2003). In the largest clinical series to date, including 1,000 patients, >75% of patients refractory to standard medical treatment achieved a clinical response to hypnotherapy, defined as a ≥ 50-point reduction in IBS symptom severity score. There were also significant improvements in extraintestinal symptoms, and anxiety and depression scores. Outcome was unaffected by bowel habit subtype (Miller et al., 2015). It is important to note that psychological therapies, but not pharmacological treatments, can lead to long-term improvement in IBS (Whorwell, 2024).

### Graduated gut-directed hypnosis

Hypnotherapy has previously only been recommended for patients with IBS when symptoms are refractory to conventional treatments (Hookway et al., 2015). A meta-analysis of RCTs showing that gut-directed hypnotherapy is one of the few treatments that performs better than a control for patients with refractory symptoms (Black et al., 2020). One of the barriers to wider scale provision of gut-directed hypnotherapy are the cost of its delivery, including time intensity, and the requirement for a trained therapist. Patients with IBS in tertiary care with severe functional limitations may require individualised hypnotherapy, with the content of sessions customised to their symptom profiles. According to the clinical experience of the author, psychotherapy in severe cases has also to target mental comorbidities, unresolved emotional conflicts and traumatic events (Häuser, 2023). In these cases, gut-directed hypnosis can be combined with psychotherapy in trance (hypnotherapy) or other psychotherapeutic methods. In addition, behavioral interventions such as graduated exposure (food, social activities) are necessary in case of an inapproriate avoindance behavior of the patient.

Patients in primary or secondary care benefit from group-delivered hypnotherapy (Moser et al., 2013). In a large, multicentre, RCT in patients with IBS in primary or secondary care, group hypnotherapy was shown to be non-inferior to individual hypnotherapy (Flik et al., 2019). In a Swedish study with 119 patients, nurse-delivered gut-directed hypnosis was as efficacous in reducing colonic, extracolonic and psychological symptoms than individual therapy (Lövdahl et al., 2022). Some of the patients of the author were able to benefit from one or two sessions of face-face gut-directed hypnosis which were recorded and used regularly by the patient afterwards. The progress of the self-management by audiotapes was monitored by email and /or video consultation. In addition, clinical outcomes via video-consultation of gut-directed hypnosis have achieved similar response rates compared with face-to-face treatment (Hasan et al., 2019).

Taking into account the limited availability of traditional and internet-delivered face-to-face psychotherapy, digital health applications (DiGAs) by app-based digital therapeutics are becoming important for IBS, too. In Germany, an electronic e-health application for IBS controlled by the German Federal Institute for Drugs and Medical Devices (BfArM) can be prescribed by physicians and the costs are reimbursed by the statutory health insurance companies. The application includes a twelve week course with education about IBS, dietary advices and psychological therapies (cognitive behavioral -based interventions and gut-directed hypnosis). The hypnosis session lasts 30 min. Daily practicing is recommended (Cara Care, 2024). No contact with a health care professionalist is possible. In a randomized controlled trial including 378 participants, 70% of the participants reported a clinically relevant reduction of IBS symptoms after the course compared to 30% in the control group which could only access the questionnaires of the app-based digital therapeutic (Weißer et al., 2023). A US study compared digital gut-directed hypnosis (GDH) with digital progressive muscle relaxation (MR) accessed via a mobile app on a smartphone or tablet in 362 patients. A similar proportion of the digital GDH (30.4%) and MR (27.1%) groups met the primary endpoint defined as ≥30% reduction from baseline in average daily abdominal pain intensity in the 4 weeks following treatment (Berry et al., 2023). Of 52 patients completing 12 sessions of remote GDH via Skype using the Manchester protocol during the COVID -19 pandemic. 27 (52%) indicated that they would have opted for remote over face-to-face GDH, regardless of the pandemic situation (Noble et al., 2022). The applicability of remote GDH only without therapeutic support might by limited by low adherence rates. Seven of 42 sessions of the Manchester protocol could be downloaded for free and 35 sessions could be purchased between June 2019 and April 2020. 2,843 patients with self-reported IBS commenced the free sessions, 1,428 (50%) purchased the app and 253 (9%) completed all 42 sessions. Users who completed the program reported clinically relevant improvements in their IBS symptoms (Peters et al., 2023). Nevertheless, digital gut-directed hypnosis could also be used as first line treatment instead of diet and pharmacological agents if preferred by the patient. The efficacy of gut-directed hypnosis is similar to guideline-recommended first line treatments of IBS. Low FODMAP-diet is recommended as first line therapy by guidelines (Lacy et al., 2021; Layer et al., 2021; Vasant et al., 2021). A controlled study demonstrated that the effects of gut-directed hypnosis were similar to those of the low FODMAP (Fermentable Oligosaccharides, Disaccharides, Monosaccharides and Polyols) for relief of gastrointestinal symptoms. Hypnotherapy was superior to the diet on psychological indices (Peters et al., 2016).

A graduated approach of gut-directed hypnosis and hypnotherapy is outlined in Table 2. The first line options might be sufficient for patients with slight IBS. In contrast, severe IBS requires a multicomponent treatment with pharmacological agents, diet and psychological therapies. The psychotherapeutic approach can combine elements of standardised gut-directed hypnosis with hypnotherapy and /or other psychological techniques such as cognitive-behavioral interventions or psychodynamic therapy (Häuser, 2023) (see Table 2).

**Table 2:** Graduated gut-directed hypnosis and hypnotherapy.

First line: standardised medical hypnosis by audiotapes or digital health applications.
Second line: standardised medical hypnosis by group therapy (personal contact or internet-delivered) or by low-frequency single therapy
Third line: standardised medical hypnosis by single therapy with personal contact and individualised hypnotherapy

### The Manchester protocol of gut-directed hypnosis

There are two standardised protocols of gut-directed hypnosis available, the North Carolina (Palsson, 2006) and the Manchester protocol (Gonsalkorale, 2006). A script of a gut-focused hypnosis and a guide with practical aspects of delivery of the Manchester protocol is available (Vasant and Whorwell, 2019). Most controlled trials have used the Manchester protocol. It was first used to treat patients with severe refractory IBS-symptoms as part of a controlled clinical trial here in Manchester, using a symptom-orientated or “gut-directed” approach (Whorwell et al., 1984) It is important to note that the Manchester protocol is package of interventions in which hypnotic interventions are embedded in consultation, education and regular daily practice by the patient with audiotapes. The protocol starts with standardised direct suggestions (medical hypnosis), but allows individualised (Ericksonian techniques) tailored to the individual psychological profile of the patient in the second part of the protocol (hypnotherapy). Patients are seen on an individual or group basis and the overall treatment package consists of an initial consultation followed by up to 12 therapy sessions, usually at weekly intervals.

The consultation provides the opportunity to establish rapport with the patient and includes by

- Obtaining a full clinical history and assessing symptoms and any contributing factors, explaining the origin of symptoms and thus offering a model for applying hypnotic intervention by giving an overview of normal gut function and the current understanding of physiological mechanisms underlying IBS symptoms, i.e., disordered motility, visceral hypersensitivity and altered processing of gastointestinal stimuli in the brain
- Reassuring the patient about hypnosis and what treatment will involve: learning mental skills and techniques to develop control over the physiological mechanisms influencing the gut that are not normally under their conscious control.

1st (and 2nd) sessions include a training in relaxation/hypnotic induction. In addition, ego-strengthening suggestions are given. From 2nd or 3rd sessions gut-directed suggestions for control and normalization of gut function, e.g., hand warmth on abdomen, imagery of a normal gut function, imagery of a healing light, imaginal rehearsal (the patient imagines him or herself in any previously feared or avoided situations—such as shopping—but now with the gut working normally), decatastropizing suggestions. During reorientation, posthypnotic suggestions are given.

The Manchester group has demonstrated in a RCT with 444 patients that six sessions of gut-directed hypnosis led to similar levels of improvement in IBS symptoms, noncolonic symptoms, anxiety, depression, and quality of life compared with 12 sessions (Hasan et al., 2021).

### Predictors of response (Manchester protocol)

With regard to bowel habit, diarrhea responds better than constipation. Women respond better than men (Whorwell, 2024). Patients who have a clear mental image of their condition and who chose a positive color to describe their mood on the Manchester Colour Wheel are more likely to respond to treatment. Interestingly, high hypnotizability measured indirectly on the Tellegen Absorption Scale did not seem to be associated with a better response to gut-directed hypnosis (Whorwell, 2024). A higher burden of gastrointestinal and extraintestinal symptoms and lower anxiety scores at baseline were predictors of response defined as ≥50-point decrease in IBS-Symptom Severity Scale or ≥ 30% reduction in pain severity scores in a post-hoc analysis of a randomised controlled trial comparing six to 12 sessions gut-directed hypnosis (Hasan et al., 2021; Devenney et al., 2024).

### Contraindications

The general contraindications for hypnosis (psychotic disorders, histrionic and borderline personality disorders and passive-receptive attitudes) (Peter and Revenstorf, 2023) are also valid for gut-directed hypnosis.

## Discussion

Gut – directed hypnosis is one of the rare sucess stories of hypnosis in medicine. It has been recommended by European and North American gastroenterology guidelines for the management of IBS. Digital health applications have enlarged the availability of gut-directed hypnosis. It is particularly interesting to note that gut-directed hypnosis relieves a wide range of symptoms associated with IBS which is in contrast to medications which often only target one symptom such as pain or bowel function. Furthermore, it often relieves the non-colonic symptoms which seldom improve with pharmacological approaches. Long-lasting effects of gut-directed hypnosis have been demonstrated in contrast to pharmacological therapies.

Gut-directed hypnosis should be regarded as part of a treatment package consisting of education, dietary manipulation, and “as necessary” medication rather than being a “stand alone” approach for moderate and severe forms of IBS. Gut-directed hypnosis can be considererd as a single therapy for mild foms of IBS. However, Whorwell and coworkers have noted that patients with very mild symptoms do not necessarily do so well with hypnotherapy presumably because the motivation for embarking on a time consuming, relatively labor intensive form of treatment is not strong enough (Whorwell, 2024).

Some authors include hypnosis into the methods of complementary and alterantive medicine (Behzadmehr et al., 2020). The US National Centre for Complementary and Integrative Health (2021) has defined CAM as “diverse medical and health care practices and products that are not presently considered to be part of conventional medicine”. However, gut-directed hypnosis is an evidence-based treatment an part of conventional medicine. Scientific hypnosis associations should fight against the inclusion of medical hypnosis as a complementary/alternative treatment within the many non-scientifically based methods of the so-called mind–body medicine (Häuser, 2022).

## Author contributions

WH: Writing – review & editing, Writing – original draft.
